# Cardiac tamponade in a newborn caused by a peripherally inserted central catheter: case report

**DOI:** 10.31744/einstein_journal/2025RC0634

**Published:** 2025-07-02

**Authors:** Carolina Tenfen, Gabriela Dominicci de Melo Casacio, Maria Fernanda Munhak da Silva, Graziely Masotti Scalabrin Barreto, Adriana Zilly, Rosane Meire Munhak da Silva

**Affiliations:** 1 Hospital Universitário do Oeste do Paraná Universidade Estadual do Oeste do Paraná Cascavel PR Brazil Hospital Universitário do Oeste do Paraná, Universidade Estadual do Oeste do Paraná, Cascavel, PR, Brazil.; 2 Universidade de São Paulo São Paulo SP Brazil Universidade de São Paulo, São Paulo, SP, Brazil.; 3 Universidade Estadual do Oeste do Paraná Cascavel PR Brazil Universidade Estadual do Oeste do Paraná, Cascavel, PR, Brazil.; 4 Universidade Estadual do Oeste do Paraná Foz do Iguaçu PR Brazil Universidade Estadual do Oeste do Paraná, Foz do Iguaçu, PR, Brazil.

**Keywords:** Infant, newborn, Infant, premature, Cardiac tamponade, Catheters, X-rays, Intensive care units

## Abstract

This article describes the occurrence of cardiac tamponade in a premature newborn after the use of a peripherally inserted central catheter at a university hospital in Brazil. Pericardiocentesis was performed, the catheter was repositioned using radiography, and minimal residual pericardial effusion was confirmed using echocardiography. The patient showed good progress and was discharged from the hospital on day 47 of life without any complications related to the event. Therefore, although adverse events may occur following the insertion of peripherally inserted central catheters, imaging examinations and exclusion diagnoses have a positive impact on clinical outcomes. This study emphasizes the importance of a multidisciplinary team for monitoring and managing adverse events and optimizing the care of critically ill newborns.

## INTRODUCTION

Peripherally inserted central catheters (PICC) are widely used in neonatal intensive care units (NICUs) because of their easy and quick insertion and ability to manage critically ill newborns.^1,2^ However, PICC insertion is an invasive procedure, and therefore may result in complications such as thrombosis, phlebitis, pleural or pericardial effusion, tamponade, and arrhythmia.^1^

The use of aseptic technique, pre-intervention surface measurements, intracavitary electrocardiogram guidance, ultrasound-guided insertion, and post-intervention radiography can reduce the risk of complications and improve outcomes.^1-3^

## CASE REPORT

**Figure 1:**
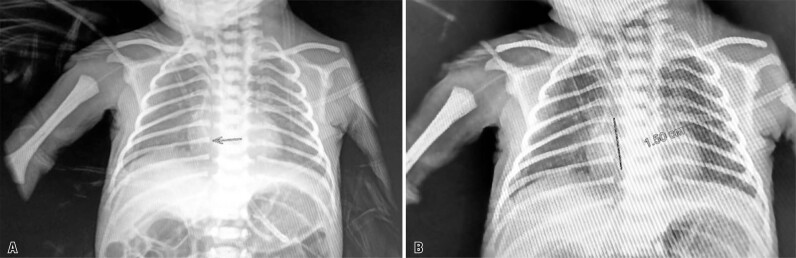
Radiographs showing the peripherally inserted central catheter shortly after insertion: A) Red arrow shows the intracardiac location of the catheter tip; B) Red line shows the extent of catheter retraction required (1.5 cm)

A premature newborn, delivered via cesarean section at 33 gestational weeks because of severe maternal preeclampsia (weight, 1,080g; Apgar scores, 6 and 7), was admitted to the NICU with a diagnosis of respiratory distress syndrome and prematurity. Initially, an umbilical venous catheter was inserted for antibiotic treatment and total parenteral nutrition (TPN); a PICC was indicated on the fourth day of life. The procedure was performed by a nurse and neonatology resident using measurements obtained from the right cephalic vein near the cubital region of the second intercostal space. Radiography performed after the procedure revealed that the catheter was located intracardially, and 1.5cm of the catheter was therefore retracted (Figure 1).

The patient was then placed in a protective neonatal nest. Non-invasive ventilation (NIV) was provided, vital signs were monitored with no abnormalities detected, and trophic feeding was resumed.

The newborn exhibited hypoxemia (72% peripheral oxygen saturation) and bradycardia (65 beats/min) 17h after PICC insertion. Since the infant was agitated and receiving NIV, a sedative (Midazolam^®^) was administered, and orotracheal intubation was performed. Subsequently, the patient entered cardiorespiratory arrest, and cardiopulmonary resuscitation was initiated along with the administration of adrenaline (seven cycles). Pericardial puncture was performed without imaging confirmation, resulting in the aspiration of 5mL of milky fluid consistent with TPN. Fluid infusion was paused and another pericardiocentesis resulted in the aspiration of an additional 7mL of fluid with the same appearance, leading to an improvement in oxygen saturation and heart rate. Peripheral venous access was established and fluid therapy was initiated.

**Figure 2:**
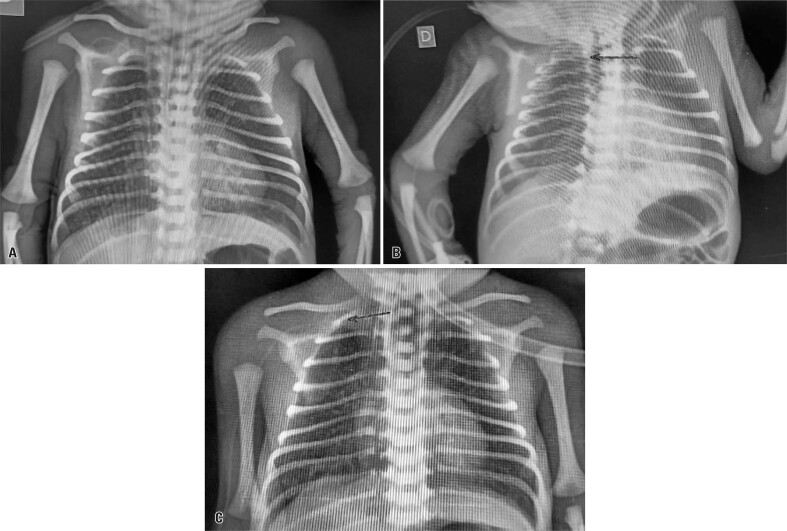
Radiographs performed after retracting the catheter by 6 cm: A) Immediately after retraction; B) 30 min after retraction; C) 24 h after retraction. The black arrow indicates the tip of the peripherally inserted central catheter

Radiography indicated the PICC was in the cardiac area. The catheter was retracted by 6cm and subsequent radiography confirmed its peripheral location (Figure 2).

Infusion via the PICC was resumed, with vasoactive drugs administered to increase cardiac output and reduce peripheral vascular resistance. Considering the diagnostic hypothesis of cardiac tamponade, echocardiography was performed, which showed minimal residual pericardial effusion 20h after the event. Extubation occurred 48h later, and vasoactive drugs were discontinued. After 96h, transcranial Doppler ultrasonography revealed no abnormalities.

The patient developed hyperemia and edema in the right upper limb and right upper thorax due to infiltration 8 days after PICC insertion. Fluid infusion was suspended and the new PICC was replaced. As the patient exhibited clinical deterioration in the form of hypoactivity, pale skin, tachycardia, and respiratory distress, the catheter was sent for culture; *Staphylococcus haemolyticus* was detected. The newborn was placed under contact isolation following the guidance of the Hospital Infection Control Commission and antibiotic therapy was adjusted. After the completion of antibiotic treatment, and with significant clinical improvement, the newborn was transferred from the NICU to the intermediate care unit on day 35 of life and discharged from hospital on day 47 of life.

This study was approved by the Research Ethics Committee of *Universidade Estadual do Oeste do Paraná* (CAAE: 69914523.9.0000.0107, # 6.081.634).

## DISCUSSION

Cardiac tamponade can occur hours or days after PICC insertion, mainly due to flushing or movement of the newborn,^3^ and should be considered when hypoxemia and bradycardia occur. To confirm the diagnosis, echocardiography should be performed to assist emergency pericardiocentesis and prevent death.^1,2,4^

Peripherally inserted central catheters migration can occur up to 72h after insertion, but is most likely within the first 24h. In addition to catheter displacement, factors such as catheter length, material, duration of TPN infusion, osmolarity, and composition of fluid therapy can contribute to a poor prognosis.^3^Intracavitary electrocardiography and ultrasonography are essential for identifying the position of the catheter without radiation exposure.^1^ Although cardiac tamponade is rare, it is associated with a high mortality rate. In the case presented here, early diagnosis by exclusion, pausing fluid infusion, and emergency pericardiocentesis directly influenced the clinical outcomes.^5,6^

The literature recommends scheduled radiography for catheter monitoring to prevent adverse events associated with PICCs. Protocols suggest imaging at 24h and 72h after insertion, and at 7-day intervals thereafter. In addition, cutting the PICC should be avoided as this can alter its tip, leading to negative clinical outcomes such as deep vein thrombosis or cardiac tamponade.^1^It is noteworthy that these protocols were in place in the NICU at the time of this event.

An international study indicated that the existence of a multidisciplinary team including neonatologists, respiratory therapists, and nurses and the standardized monitoring of newborns with PICCs reduced the requirement for catheter manipulation and radiography.^7^

In the case presented here, PICC removal was required after 8 days; however, the literature suggests that PICC removal may not be necessary after pericardiocentesis, as it exposes the newborn to greater manipulation.^8^

## CONCLUSION

Although peripherally inserted central catheters use is recommended for neonates, with the appropriate imaging examinations to confirm catheter positioning, rare events can still occur, particularly in premature infants. Therefore, the presence of an attentive and trained professional team is necessary to intervene promptly in emergency situations, as is the rigorous monitoring of catheter positioning based on robust protocols and using appropriate equipment. Following this event, double-checking/visualization of catheter positioning was adopted in our neonatal intensive care units to improve peripherally inserted central catheters position monitoring.
